# Digital Age Transformation in Patient-Physician Communication: 25-Year Narrative Review (1999-2023)

**DOI:** 10.2196/60512

**Published:** 2025-01-16

**Authors:** Mingming Song, Joel Elson, Dhundy Bastola

**Affiliations:** 1 University of Nebraska at Omaha Omaha, NE United States

**Keywords:** health communication, health IT, patient empowerment, shared decision-making, patient-physician relationship, trust

## Abstract

**Background:**

The evolution of patient-physician communication has changed since the emergence of the World Wide Web. Health information technology (health IT) has become an influential tool, providing patients with access to a breadth of health information electronically. While such information has greatly facilitated communication between patients and physicians, it has also led to information overload and the potential for spreading misinformation. This could potentially result in suboptimal health care outcomes for patients. In the digital age, effectively integrating health IT with patient empowerment, strong patient-physician relationships, and shared decision-making could be increasingly important for health communication and reduce these risks.

**Objective:**

This review aims to identify key factors in health communication and demonstrate how essential elements in the communication model, such as health IT, patient empowerment, and shared decision-making, can be utilized to optimize patient-physician communication and, ultimately, improve patient outcomes in the digital age.

**Methods:**

Databases including PubMed, Web of Science, Scopus, PsycINFO, and IEEE Xplore were searched using keywords related to patient empowerment, health IT, shared decision-making, patient-physician relationship, and health communication for studies published between 1999 and 2023. The data were constrained by a modified query using a multidatabase search strategy. The screening process was supported by the web-based software tool Rayyan. The review methodology involved carefully designed steps to provide a comprehensive summary of existing research. Topic modeling, trend analysis, and synthesis were applied to analyze and evaluate topics, trends, and gaps in health communication.

**Results:**

From a total of 389 selected studies, topic modeling analysis identified 3 primary topics: (1) Patient-Physician Relationship and Shared Decision-Making, (2) Patient Empowerment and Education Strategies, and (3) Health Care Systems and Health IT Implementations. Trend analysis further indicated their frequency and prominence in health communication from 1999 to 2023. Detailed examinations were conducted using secondary terms, including trust, health IT, patient-physician relationship, and patient empowerment, derived from the main topics. These terms clarified the collective impact on improving health communication dynamics. The synthesis of the role of health IT in health communication models underscores its critical role in shaping patient-centered health care frameworks.

**Conclusions:**

This review highlights the significant contributions of key topics that should be thoroughly investigated and integrated into health communication models in the digital age. While health IT plays an essential role in promoting shared decision-making and patient empowerment, challenges such as usability, privacy concerns, and digital literacy remain significant barriers. Future research should prioritize evaluating these key themes and addressing the challenges associated with health IT in health communication models. Additionally, exploring how emerging technologies, such as artificial intelligence, can support these goals may provide valuable insights for enhancing health communication.

## Introduction

### Background

Technological advancements have transformed patient-physician communication. A fundamental aspect of this transformation is through patient empowerment, which encourages individuals to manage their health proactively according to their personal needs, thereby improving health care outcomes [1]. The adoption of patient empowerment practices and broader access to health information from various sources such as online platforms have shifted from a traditional 1-way communication model to a patient-centered approach that emphasizes collaborative shared decision-making [2-4]. This shift promotes active patient engagement, encouraging them to articulate their needs, preferences, and values within the health care process [5,6]. Additionally, today’s health communication is often enhanced by technological advancements that provide patients with extensive health information and tools [7,8]. Telehealth, mobile health (mHealth), patient portals, and recently adopted artificial intelligence (AI) techniques have expanded patients’ access to health care and health-related data [9,10]. Telehealth platforms, for example, have made medical consultations more accessible, allowing patients to communicate with health care providers remotely [10,11]. This convenience helps patients actively participate in consultations and make informed decisions about their treatments. Similarly, electronic health records (EHRs) facilitate a continuous flow of information between different health care providers, ensuring that every provider involved in patients’ care has access to the same comprehensive data [12]. By 2021, 88% of US office-based physicians had adopted EHR [13]. This widespread adoption ensures that providers have timely and accurate patient data, which is crucial for tailoring treatment plans to individual needs and preferences. Additionally, patient portals give patients 24/7 access to their health records, test results, and direct communication with their health care providers [14]. This transparency encourages patient engagement and fosters active discussion about care plans, ultimately enhancing the effectiveness of patient-physician communication. Despite these benefits, the availability of online health information also introduces risks, such as the potential exposure to misinformation [15-17]. Misinformation can undermine the trust necessary for effective health care relationships, which is critical because trust influences patients’ willingness to follow medical advice and maintain open communication with their providers. For instance, the impact of social media on these relationships significantly varies based on the accuracy of the information and the preexisting relationship between patient and physician, which can range from positive to negative [18].

Today’s diverse health information technology (health IT) landscape further underscores the need for a refined health communication model that effectively leverages digital tools to enhance patient empowerment across various health care settings [19,20]. While the concepts of patient empowerment and patient-centered care are frequently discussed, their integration into health communication models is still not fully addressed. As digital solutions become standard in health care, it is increasingly important to understand how patient relationships, empowerment, and health IT integrate and interact within health communication models. This extensive review explores essential topics of health communication that fit today’s digital age and investigates their associated dynamics to identify mechanisms that facilitate effective health communication between patients and physicians.

### Aim and Objectives

The overarching aim of the research is to investigate the essential elements for inclusion in a refined health communication model to enhance patient-physician communication and shared decision-making in the current digital age. Using the PICo framework (Population, Interest, and Context) [21], this study specifies the population as physicians and patients, the interest as key elements for effective communication models, and the context as the impact of health IT on health communication. Particularly, the first objective is to identify and analyze the key topics and factors that have influenced health communication in the past 25 years and should be incorporated into effective health communication models. The second objective involves trend analysis to explore the dynamics and associations among those key topics and factors identified in the first objective. Finally, the last objective focuses on synthesizing the impact of health IT on patient-physician interactions and its subsequent effects on the evolution of health communication models.

However, unlike systematic reviews, this extensive review does not adhere to strict protocols for study selection and data extraction; instead, we aim to provide a comprehensive synthesis of available evidence to inform future research and practice in the health communication model. In the subsequent sections of this paper, we will provide an overview of the methodologies used to explore and analyze essential factors in health communication, including detailed descriptions of our data selection, screening processes, and analysis techniques such as topic modeling and trend analysis.

## Methods

### Search Strategy and Eligibility Criteria

To answer our research question and meet our study objectives, we conducted an extensive literature review using the web-based software tool Rayyan for screening, which enabled us to efficiently filter through a large amount of research [22]. The review and reporting process followed the guidelines provided by Rowley and Slack [23,24], ensuring a rigorous approach to organizing the collected literature and presenting the findings in a structured and coherent manner (Multimedia Appendix 1).

Multiple electronic databases including PubMed, Web of Science, Scopus, PsycINFO, and IEEE Xplore were searched to ensure broad coverage of the literature. Each database was chosen for its specific strengths and relevance to various dimensions of health communication. PubMed was selected for its comprehensive coverage of biomedical literature, essential for addressing medical aspects of health communication. Web of Science and Scopus were chosen for their multidisciplinary scope, enabling the examination of trends across diverse fields. PsycINFO was included for its focus on psychological literature, key for exploring aspects of patient empowerment and decision-making processes. Finally, IEEE Xplore was included to access cutting-edge research on technological innovations in communication tools, critical for understanding the development and application of digital solutions in health care.

While digital tools and patient involvement are reshaping health communication, we carefully selected keywords representing 4 different categories related to health communication, including patient participation (MeSH [Medical Subject Headings] term), decision-making, shared (MeSH term), technology (MeSH term), and physician-patient relations (MeSH term) to guide our literature selection. These keywords were used along with Boolean operators (AND or OR) to capture diverse studies that allow us to examine the complex dynamics between the interactions of patients and physicians. To ensure a robust and precise alignment with our research objectives, we thoroughly reviewed our keywords and relevant key terms using the MeSH database and implemented a multidatabase search strategy [25]. This approach allowed us to craft a query that effectively targets the most relevant literature across various platforms, enhancing the breadth and depth of our literature review. Each query was adjusted slightly depending on the database, covering the period from 1999 to 2023: ((communication) OR (health communication model)) AND ((decision making*) OR (decision making, shared)) AND ((patient participation*) OR (patient participation/methods) OR (patient empowerment) OR (patient engage*) OR (patient involve*) OR (Patient-Centered Care)) AND ((physician-patient relations*) OR (Trust) OR (physician-patient trusted relationship)) AND ((decision support techniques) OR (technology) OR (health informatics) OR (Patient Preference) OR (information systems*) OR (health information systems))

The selection process for the literature review was carefully designed to comprehensively cover studies on patient-physician interactions within the digital health framework. We included studies that (1) examined these interactions specifically with health care technologies; (2) aligned with the principles of shared decision-making or patient-centered care; (3) focused on cancer or chronic diseases, given their significant implications for patient communication; and (4) were published within the designated time frame of 1999-2023 to capture the evolution of health communication practices in the past 25 years. To maintain the quality and relevance of the studies reviewed, exclusions were made based on (1) language, excluding non-English articles to ensure consistency in data interpretation; (2) publication type, omitting reports, conference papers, abstracts, letters, or feature articles, which lack peer-reviewed validation; and (3) the absence of significant patient participation or empowerment, a key focus in evaluating effective communication strategies. This structured evaluation ensures our review meets high scholarly standards, offering a solid foundation for understanding the changing dynamics of health communication with emerging health care technologies.

### Data Analysis

#### Topic Modeling and Visualization

All our analysis processes for this research were applied in R (R Foundation). Initially, we used the “tm” package to preprocess the text, which involved transforming all characters to lowercase, removing punctuation, numbers, and custom stopwords to ensure a clean and relevant data set. Following preprocessing, we constructed a Document-Term Matrix from the cleaned text to remove sparse terms and enhance both computational efficiency and model accuracy. To assess the coherence and effectiveness of the model, we utilized the “ldatuning” package and determined 3 optimal numbers of topics based on coherence scores, each representing a distinct thematic cluster within our data. Subsequently, we applied latent Dirichlet allocation using the “topicmodels” package to identify the most frequently discussed terms and concepts within the abstracts of the collected studies.

To aid in the interpretation of these topics, we visualized the topics using the “LDAvis” package. This advanced visualization tool allowed us to create interactive 2D scatterplots and bar plots of the topics. By adjusting the lambda parameter from 0 to 1, we were able to explore both the unique and frequently occurring terms within each topic, enhancing our understanding of the thematic relevance and distinction.

#### Trend Analysis and Secondary Topic Exploration

In our analysis framework, we distinguished between primary and secondary topics to thoroughly map the landscape of health communication. The primary topics, which center on health communication between patients and physicians, were categorized by topic modeling, serving as the overarching theme of our study. Accordingly, each paper was assigned a topic number by identifying the dominant topic based on the highest probability derived from the latent Dirichlet allocation model. These topics will be utilized in our trend analysis like topic distribution over time to examine the evolving dynamics within health communication, providing insights into how these themes have developed in the past 25 years.

To effectively represent the broad themes identified through topic modeling, we have selected specific secondary terms that closely relate to these primary topics. These terms are chosen for their frequent appearance within the context of identified primary topics and their ability to precisely capture the core ideas of each topic. Once identified, these terms are used as critical filters to refine our data set individually, ensuring that only those papers most relevant to each secondary topic were included. This step is critical as it allows us to apply the themes for detailed trend analysis and in-depth synthesis, particularly suitable for methodologies such as UpSet plotting, where the identification of overlaps and intersections between sets is necessary.

#### Impact Synthesis

To achieve our third objective, synthesizing the impact of health IT on patient-physician interactions and its subsequent effects on the evolution of health communication models, our final analytical phase involved a rigorous review process focusing on studies that exemplify the practical integration of health IT within dynamic health communication frameworks. These frameworks are designed to actively involve both patients and physicians, ensuring that technology enhances their communication. From the literature used for data analysis, we further narrowed the focus to a specific analysis centered on health IT and its application in health communication. We strictly excluded studies that (1) did not incorporate health IT as an integral component of patient-physician communication, (2) solely evaluated technology design without practical health care application, or (3) lacked a structured communication model. This exclusion criteria allowed us to concentrate on research that provided clear insights into effective health IT integration.

This comprehensive and structured methodology ensures that our analysis not only adheres to rigorous academic standards but also provides insightful conclusions that enhance our understanding of the dynamic field of health communication in the digital age.

## Results

### Outcomes

In this section, we first present the outcomes of our data selection and screening process. Through query adjustment and refinement, removal of duplicates, and application of strict selection criteria, we narrowed our focus to 389 papers. These articles were then utilized in topic modeling, providing a foundation for further analysis.

In our topic modeling analysis, 3 major topics were identified, each closely related to health communication. This structured framework allowed for deeper exploration of the data and set the stage for subsequent analyses. Building upon the results of topic modeling, we conducted trend analysis to explore the dynamics and associations between significant elements such as health IT, patient-physician relationships, trust, and patient empowerment. The distribution of these topics over time and the interrelations among these key elements were visualized using topic distribution graphs and UpSet figures, offering a clear depiction of evolving trends.

Finally, our study synthesized findings on the role of health IT in patient-physician interactions, highlighting this as the key aspect of our third objective. This phase highlighted how technological advancements have been integral in shaping modern health communication models and identified areas for further development to facilitate enhanced interactions between patients and health care providers.

### Data Selection and Screening

**Figure 1:**
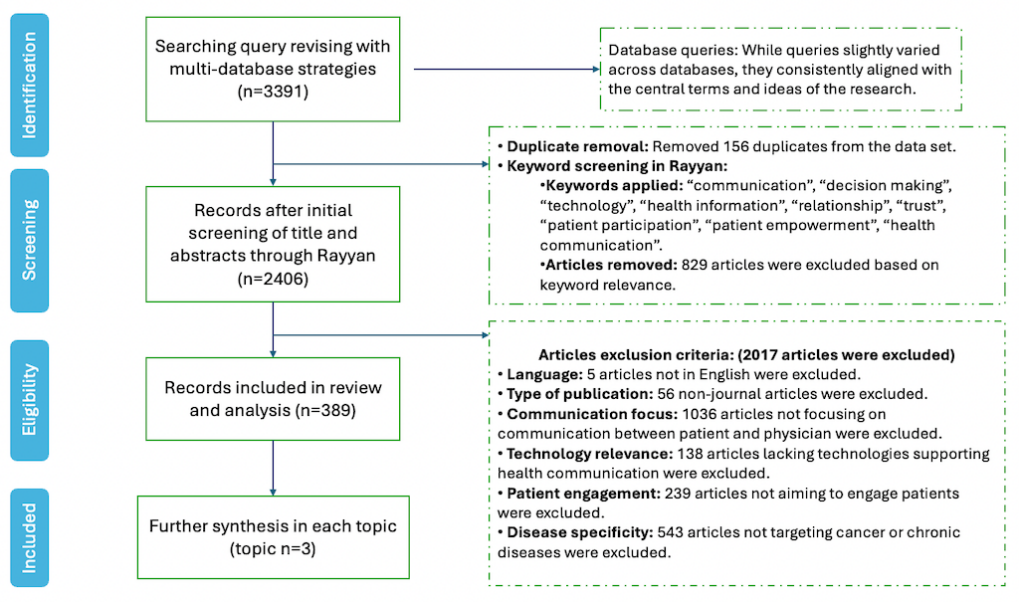
The diagram illustrates the selection process (identification, screening, eligibility assessment and inclusion) of this review study.

The review process initially identified and collected 3391 articles through multiple databases, as illustrated in Figure 1. Following the initial search, the titles and abstracts of these articles were screened using Rayyan. This initial screening narrows down the data to 2406 articles by filtering out those that clearly do not meet our preliminary inclusion criteria and 156 duplicated articles, ensuring the uniqueness of each study included in the analysis. The remaining articles were subjected to a rigorous screening process based on specific exclusion criteria, including language (English only), publication period (1999-2023), type of publication (journal articles only), and content relevance (articles focusing on communication involving technology and patients, specifically within the contexts of cancer or chronic diseases). This process effectively narrowed our focus to 389 articles that were highly relevant to the core topics of health communication, enabling us to explore the dynamics and associations between significant elements.

### Topic Modeling Analysis

To address objective 1, identifying and analyzing key topics that influence health communication, we conducted a topic modeling analysis on the selected 389 articles. This analysis successfully identified and characterized 3 main topics, which explain the underlying structure of health communication and reveal distinct themes that enhance patient-physician communication in the digital age.

**Figure 2:**
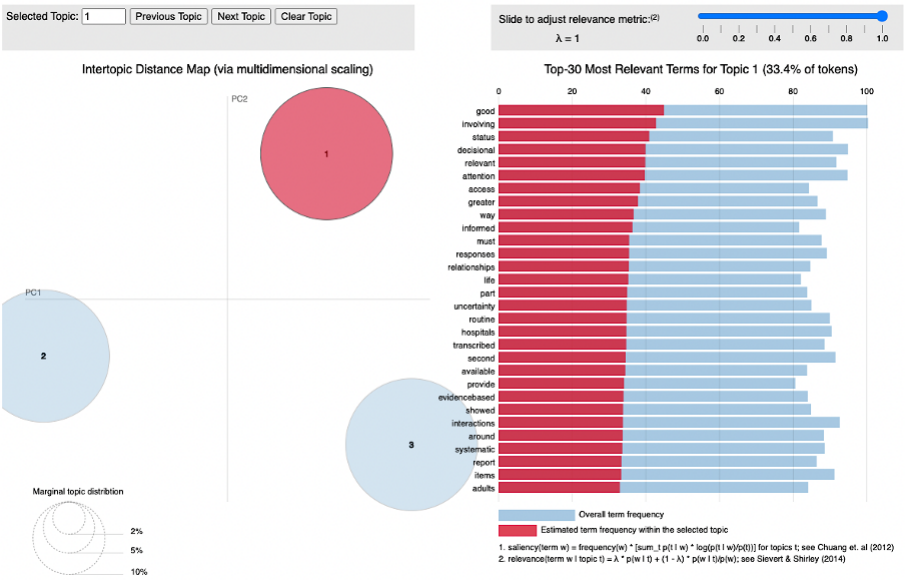
A 2D scatterplot and a bar plot illustrating the distribution and relevance of topics identified from the topics modeling analysis using LDAvis. Each circle in the scatterplot represents a topic, while the bar plot displays the most relevant terms for topic 1 as an example here.

For the visualization of our topic modeling results, the scatterplots in Figure 2 explain how principal component 1 and principal component 2 capture the directions in which the variance in the topic distribution is most pronounced. As the topics, represented by the circles in the scatterplot, are far apart from each other, they are more likely to have a unique set of terms in each topic. To gain a deeper insight into the underlying structure, a fine-tuning option with lambda was used to adjust the relevance and frequency of terms in each topic. Adjusting the lambda value from 0 to 1 shifted the relevance of terms between their frequency in the data set and their uniqueness within the topic (ie, with a low lambda value the topics appeared somewhat similar, whereas higher values provided a clearer distinction between the topics). Further details on topics 2 and 3 are provided in Multimedia Appendix 2.

With distinct themes central to health communication, we carefully named and described individual topics by their unique set of keywords. These topics and their descriptions are summarized in Table 1.

**Table 1:** The 3 topics categorized from topic modeling results and selective top keywords.

Topic	Label	Keywords
Topic 1	Patient-Physician Relationship and Shared Decision-Making (n=119)	“good,” “status,” “decisional,” “access,” “attention,” “greater,” “informed,” “must,” and “relationships”
Topic 2	Patient Empowerment and Education Strategies (n=142)	“type,” “engage,” “evaluated,” “measured,” “following,” “topics,” “electronic,” “educational,” “older,” and “diagnosis”
Topic 3	Health Care Systems and Health IT Implementations (n=128)	“association,” “reduce,” “clinician,” “national,” “provided,” “models,” “digital,” “age,” and “interactions”

Topic 1, labeled *Patient-Physician Relationship and Shared Decision-Making*, describes the quality and process of health care delivery focusing on informed and shared decision-making and patient-physician relationships. In health communication, this topic can indicate how patients’ access to information and the quality of the patient-physician relationship contribute to effective communication and shared decision-making.

Topic 2, labeled *Patient Empowerment and Education Strategies*, focuses on educational strategies and evaluation methods in health care, emphasizing the utilization of health IT and targeting aging populations. In health communication, this topic can explore how empowering patients through education and supporting them with digital tools improves communication and healthcare outcomes.

Topic 3, labeled *Health Care Systems and Health IT Implementations*, is specifically related to health care systems and technological implementations. In health communication, it can explore how health care policies and technological implementations facilitate effective communication and enhance models by guiding interactions between patients and physicians.

The topic modeling results have strengthened and consolidated the categories of our designed searching queries, combining “patient-physician relationship” and “shared decision-making” into a single focused topic. Additionally, it has emphasized the importance of educational strategies for patient empowerment and the critical role of health IT implementation in health care systems.

Based on these findings, we carefully selected our secondary terms “patient-physician relationships,” “patient empowerment,” and “health IT” to reflect the core themes identified in the primary topics. The patient-physician relationship was explored using terms such as “patient-physician relationship,” “relationship,” “shared decision-making,” and “patient-centered care.” In the case of patient empowerment, we used keywords such as “empowerment,” “participation,” “engagement,” “involvement,” and “self-management.” Health IT was represented by terms such as “technology,” “online platform,” “EHR,” “telehealth,” “mHealth,” “digital,” “informatics,” “patient portal,” “wearable device,” “telemedicine,” “remote,” and “information system.”

A further manual review was conducted by the first author and closely monitored by the project lead to ensure consistency and accuracy in the analytical approach. Beyond the identified topics, trust emerged as a significant theme within health communication and a frequently discussed topic across various topics analyzed in the study. Topic 1 related to patient-physician relationship and shared decision-making has 42 trust-related articles out of 119 (35.3%), topic 2 on patient empowerment and educational strategies has 45 out of 142 (31.7%), and topic 3 concerning health care systems and health IT implementations has 40 out of 128 (31.3%). These findings highlighted the need to include trust as an additional secondary element in health communication and further explore it in the rest of the analysis to investigate its association with health communication and the interplay among other key factors. Therefore, we utilized terms such as “trust,” “physician-patient trust,” “medical trust,” “health care trust,” and “trust in health information” to represent trust in our following analysis. An additional observation from this review indicates that the topic of trust can include a broad range, extending beyond patient-physician trust to potentially include trust in health care systems, digital tools, and health information.

### Trend Analysis

To address objective 2, exploring the dynamics and associations among key topics and factors identified through topic modeling, our trend analysis was structured into 2 main sections. Initially, we used a bar plot to visually represent the frequency and prominence of each primary topic from 1999 to 2023. Following this, we used an UpSet plot to further explore the contributions and intersections of secondary terms that were carefully selected to represent primary topics over time.

**Figure 3:**
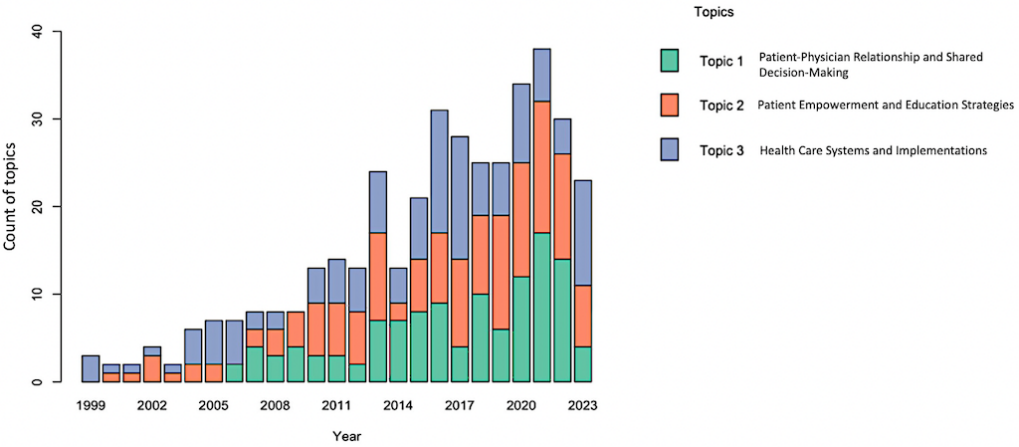
Bar plot illustrating the annual distribution of papers categorized by their relevance to 3 identified topics from topic modeling with different colors. The bar plot provides a visualization of how the distribution and frequency of each topic within health communication have evolved from 1999 to 2023.

The first section of the trend analysis focused on the primary topics that we found through topic modeling within health communication. By analyzing the topic distribution and assigning a unique topic number ranging from 1 to 3, we tracked the frequency of each topic over the years (1999-2023) with a bar plot (Figure 3).

Topic 1 (green), Patient-Physician Relationship and Shared Decision-Making, did not emerge in the literature until 2006. It shows a notable presence across the years with some fluctuations, specifically with a significant peak in 2021 (frequency 17, mean 5.8).

Topic 2 (orange), Patient Empowerment and Education Strategies, reveals a steady increase in frequency over the years, emphasizing a consistent rise in research interest and practical applications (mean 4.92).

Topic 3 (blue), Health Care Systems and Health IT Implementations, exhibits an upward trend, with notable peaks in 2016 (frequency 14) and 2023 (frequency 12), compared with a mean frequency of 5.16.

**Figure 4:**
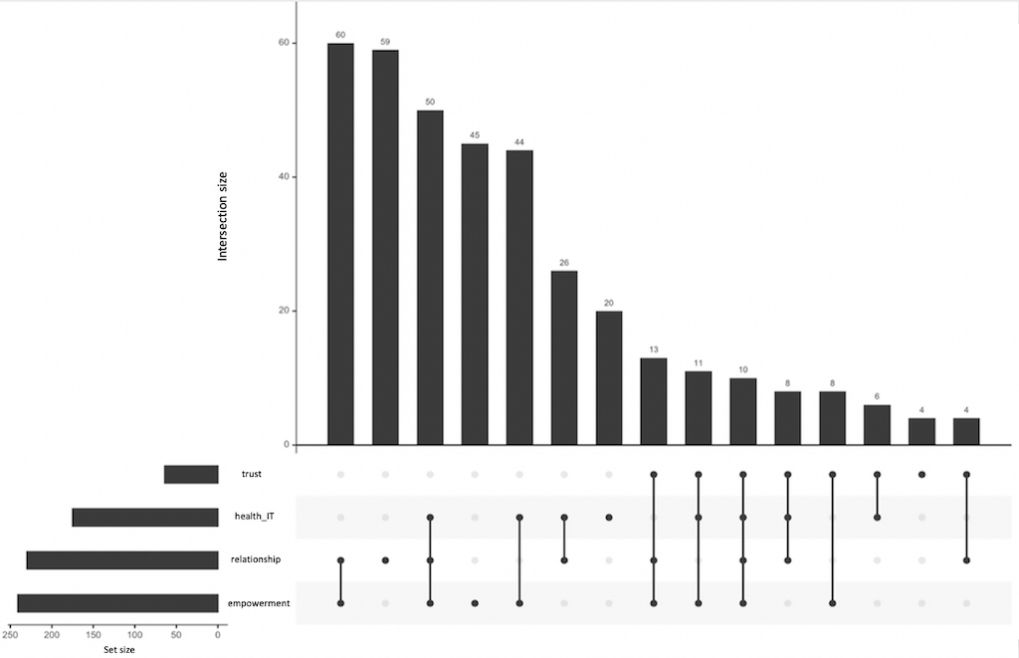
UpSet figure showing the key factors of health communication, including patient empowerment, patient-physician relationship, health IT, and trust, which are represented in 4 different sets individually. The bar chart on the left shows the size of each set, which corresponds to the number of articles that contains any factors among our key factors. The main bar chart on the top represents the size of intersections between different sets within health communication. Each bar's height indicates how many elements are common to the individual sets connected below by dots and lines in the matrix. The matrix of intersections at the bottom has rows labeled with the names of the 5 sets and columns represented by dots under each set name. A filled dot indicates that the set involves the intersection corresponding to that column.

In the second section of our trend analysis, we utilized an UpSet plot (Figure 4) to explore the associations among the secondary terms within health communication. This analysis specifically focuses on patient empowerment, patient-physician relationships, health IT, and trust in the field of health communication.

In our UpSet figure analysis within the scope of health communication, “patient empowerment” emerges as the most prominent set, as indicated by the largest bar on the left side of the plot. It is closely followed by “patient-physician relationship” and “health IT,” both highlighting their critical roles in the dynamics of health communication. The topic of “trust” shows a slightly lesser focus. Intersections between these sets reveal notable overlaps, with the largest interaction between “patient empowerment” and “patient-physician relationship” under health communication. This intersection reflects key themes of patient-centered care that involve communication strategies, empowerment, and interpersonal relationships. Reinforced by its presence in the second largest intersection with health communication, the “patient-physician relationship” further emphasized its critical role. This continual involvement across different sets highlights that the patient-physician relationship is central to discussions in health communication, further emphasizing the critical need for strong interpersonal foundations between patients and physicians in the patient-centered care framework. Similarly, the interaction involving “health IT,” “patient-physician relationship,” and “patient empowerment” under health communication illustrates an increasing integration of technology in empowering patients to facilitate effective health communication. Finally, although “trust” is addressed in a relatively smaller number of articles, its presence in complex interactions with all other factors highlights its foundational importance and influence, suggesting potential for deeper exploration to uncover its broader impacts on health communication.

### Synthesis of Health IT’s Impact on Patient-Physician Communication Models

To address our third objective, synthesizing the impact of health IT on patient-physician interactions, we conducted a detailed review based on our secondary research terms of health IT. This process led to the selection of 29 pivotal papers for detailed analysis, the findings of which are thoroughly detailed in Table 2.

These papers collectively provide a synthesized overview highlighting the specific health communication models or frameworks employed, the variety of health IT tools utilized, their impacts on patient empowerment and engagement, and the challenges or barriers encountered. The analysis revealed several key themes to not only map out the direct effects of health IT in health communication but also indicate practical applications and recommendations aimed at overcoming the identified challenges and amplifying the effectiveness of health communication strategies, which are summarized in Textbox 1.

**Table 2:** Impacts of health information technology on patient-physician communication models.

Study	Health communication model/framework	Health information technology tools used	Impact on patient empowerment/engagement	Challenges/barriers	Better practices/recommendations
Heesen et al [26]	SDM^a^ guided	Evidence-based decision aids and educational programs	Enhances patient participation in treatment decisions	Balancing patient autonomy with a lack of initial knowledge	Continue development of comprehensive patient education programs
Sillence et al [27]	Informed SDM guided	Internet information	Empowers patients by enhancing informed decision-making and improved communication with physicians	Poorly designed websites can lead to rejection of quality content	Well-designed personalized websites with credibility
DuBenske et al [28]	SDM guided	Interactive health communication system	Provides information, communication, coaching resources, and a symptom-tracking system; bridges communication gaps among patients, families, and physicians	User diversity in format preferences and technology comfort, physician buy-in, and integration with existing medical systems	Conduct needs assessment, involve stakeholders, accommodate diverse technology preferences, ensure clinician training and buy-in, and integrate with existing systems
Jimbo et al [29]	SDM guided	Decision aid	Incorporates interactive personal risk assessment and preference clarification tools; facilitates informed decision-making and screening adherence; improves decision quality	Balancing the complexity of decision aids with user-friendly designs	Ensure decision aids are intuitive and support real-time interaction to enhance decision-making
Nazi [30]	SDM guided	PHRs^b^: secure messaging and PHR portal	Improved access to health information; enhances communication, patient self-reporting, and provider-patient relationships	Integration with clinical workflow, training, and support for health care professionals	Ensure PHR systems align with clinical practices, provide adequate training, and actively engage clinicians in PHR use
Wilkes et al [31]	SDM guided	EHRs^c^ and decision support tools	Facilitates informed and value-concordant or personalized decisions; enhances the quality of prostate cancer screening decisions	Complexity of discussions and emotional and informational barriers	Utilize EHRs for SDM support, integrate decision aids, and adjust reimbursement to encourage SDM
Smits et al [32]	Not specific; SDM guided	Web-based information tool	Enhances patient and family engagement by providing tailored information; a comprehensive approach to developing patient-centric informational tools	Balancing diverse patient preferences and literacy levels	Follow the DoTTI^d^ framework for iterative development and stakeholder engagement throughout the process
Roter 2015 [33]	SDM guided	EMR^e^ and secure patient portals	Facilitates a new model of patient-physician collaboration with high transparency	Distraction of physicians by EMR use during consultations	Implement training using a practices inventory and provide patients with full access to their EMR
Lafata et al [34]	PCC^f^	EHRs, HRA^g^ instruments, and patient reminder lists	Facilitates patient involvement and personalized care; EHRs for information management, HRAs for health risk assessment, and reminder lists for patient preparation	EHR use may extend visit duration and impact preventive service delivery and variability in tool effectiveness	Monitor tool impacts rigorously and tailor tool use to individual patient and practice needs to optimize outcomes
Bruinessen et al [35]	SDM guided	Web-based communication tool (PatientTIME)	Enhances patient self-efficacy in clinical communication	Ensuring the intervention effectively translates into measurable confidence improvements	Continuously refine and evaluate the intervention based on patient feedback and trial results and focus on integrating practical communication strategies
Olomu et al [36]	SDM guided	Decision aids	Increases medication adherence, enhances patient satisfaction with communication and confidence in health care decisions	Ensuring consistent implementation and adaptation in varied clinical settings	Further develop and test the program to evaluate its broader applicability, effectiveness, and cost-efficiency
Lussier et al [37]	PCC	e-Learning platforms	Enhances patient engagement in communication	Ensuring patient access and familiarity with web-based tools	Integrate web-based educational tools into routine practice and encourage patients to participate as part of their care
Hakone et al [38]	SDM guided	Personalized health risk communication tool	Enhances patients’ understanding of personalized risk and treatment options; improves patient decision-making capability	Balancing complexity and clarity in visual risk communication and emotional distress impact	Carefully design visual tools and narratives to meet patient needs and consider emotional states and numeracy levels
Renzi et al [39]	PCC	eHealth and self-management platform	Enhances self-management and patient empowerment in prostate cancer care with a web-based platform for patient education and management	Communication gaps postdiagnosis and postsurgery, and lack of support in self-management	Strengthen health care provider-patient communication, incorporate social support into patient care, and personalize care approaches from diagnosis through treatment
Lu and Zhang [40]	Not specific; SDM guided	Online health communities	Positive impact of physician-patient communication on compliance mediated by perceived information quality, decision-making preference, and concordance	Quality control of online health information and the gap between perceived and actual quality of information	Strengthen the management of online health information, foster open discussions and emotional connections within OHCs^h^, and ensure high-quality health information and communication
Yu et al [41]	SDM guided	Online decision aid	Facilitates patient participation in decision-making, strengthens physician-patient relationships	Conflicting agendas between patients and providers and integration of new tools into routine practice	Utilize SDM and goal-setting tools to resolve conflicts and align treatment goals and ensure tools are flexible and respect professional identities
Kondylakis et al [42]	Data-driven communication	ICT^i^ platforms	Enhances patient engagement and personalized cancer care management	Patient engagement, interoperability, knowledge management, and trust in technology	Increase trust and stakeholder engagement, ensure interoperability of ICT systems, and enhance knowledge management in ICT platforms
Hazara et al [43]	PCC	Web-based portals	Enhances patient participation, empowerment, and self-management in their health care; web-based patient portal allowing access to EHRs and communication with health providers	Barriers to widespread portal use	Enhance portal features, learn from other portal implementations, and integrate portal use more effectively in health care delivery
Gilljam et al [44]	SDM guided; Shier’s model of participation	Digital communication tools (Sisom for children)	Enhanced participation in health care; increased engagement and direct communication toward children using Sisom; children felt listened to and could express views	Lack of validation and possible variations in engagement not systematically measured	Use randomized control designs for further validation, focus on eHealth’s effects on health outcomes and cost-effectiveness, and consider expanding the use of Sisom or similar tools in pediatric settings
van Velsen et al [45]	SDM guided; active and collaborative patient-physician communication	Online patient portal	The study explored the dimensions of trust that might influence patient engagement but found no direct effect on usage	Trust does not directly influence technology usage, indicating possible other factors affecting eHealth service adoption	Assess multidimensional trust factors separately to understand their influence on eHealth adoption; further research is needed to explore why trust does not directly translate to usage
Dimitri et al [46]	Not specific; SDM guided	eHealth platforms and artificial intelligence predictions	Enhanced patient and caregiver communication, improved access to educational resources, and better medical and psychological support; use of connected devices for monitoring and artificial intelligence for predicting treatment adherence	Implementation in specific clinical settings, ensuring data privacy and security, and integration into existing clinical workflows	Involve clinicians and patients in technology integration, provide robust evidence of value and utility, address data privacy concerns
Schubbe et al [47]	Not specific; SDM guided	EHRs, email, and patient portals	Improved patient involvement in decision-making, did not lengthen consultation times, and normalized in routine care	Barriers to routine integration due to clinical and organizational factors	Adapt conversation aids to fit clinical workflows, ensure flexibility in their use, and consider patient preferences and characteristics for tailored communication approaches
Kim et al [48]	PCC	Digital health communication intervention with tailored messages based on local cultures	Enhanced family communication of genetic results and decision-making	Navigating cultural and linguistic differences in genetic education across countries	Use of existing digital platforms to facilitate culturally sensitive communication and adaptation of tools rather than creating new ones
Lu and Zhang [49]	Not specific; focusing on the effectiveness of communication	eHealth and online health communities	Increased patient adherence via enhanced eHealth literacy and positive correlation between eHealth literacy and patient adherence through improved communication and information quality	Addressing gaps in the perceived and actual quality of information	Strengthen management of information quality, develop user-friendly features, and educate patients to improve eHealth literacy
Schubbe et al [47]	SDM	Mobile health	Facilitates enhanced shared decision-making, reduces decisional conflict and regret, and improves communication quality	Addressing the specific needs of minority populations and integrating decision partners in the decision-making process	Use of community patient navigators to provide support during decisions, leverage mobile health for real-time data and engagement, and facilitate provider-patient-decision partner communication
Liu et al [50]	PCC	PAEHR^j^ systems	Improves health self-efficacy and patient-centered communication by facilitating access to health information and communication	Lack of direct association between PAEHR portal use and health outcomes	Integrate PAEHR systems to provide PCC, enhance health self-efficacy, use portals to facilitate patient engagement in health care decisions, and ensure the accessibility of PAEHR portals to improve patient health outcomes
Yılmaz et al [51]	Enhanced patient participation communication	Health Communicator (eHealth tool)	Intended to fulfill unmet informational and psychosocial support needs; multilingual eHealth tool tailored to specific ethnic groups	Patients’/survivors’ low health literacy, cultural taboos, lack of trust in health care, and perceived complexity of the tool	Ensure simplicity and ease of use in eHealth tools, involve both patients and professionals in the design process, and provide training and support for new technologies
Grynne et al [52]	Effective interpersonal communication	Digital information tool (Digi-Do)	Enhanced patient knowledge, competence, and motivation; iterative access to reliable health information, and tailored digital information tools; as well as improved preparedness before, during, and after radiation therapy	Unmet information needs, varying levels of health literacy, and reliance on non–health care professional sources	Tailor digital information tools to patient’s health literacy level, provide iterative access to reliable information, and complement interpersonal communication
Orstad et al [53]	SDM	Decision aids	Enhanced information exchange needs and addressed patient/clinician relationships and decision-making process	Continuity of patient-clinician relationships, barriers to information exchange, uncertainty in decision-making, and autonomy negotiation	Comprehensive decision support interventions, communication training on scientific/existential uncertainty, and organizational changes for better continuity and dialogue time

^a^SDM: shared decision-making.

^b^PHR: personal health record.

^c^EHR: electronic health record.

^d^DoTTI: Design and Development, Testing Early Iterations, Testing for Effectiveness, Integration, and Implementation.

^e^EMR: electronic medical record.

^f^PCC: patient-centered communication.

^g^HRA: health risk appraisal.

^h^OHC: online health community.

^i^ICT: information and communication technology

^j^PAEHR: patient-accessible electronic health record.

**Key themes identified in the analysis.**

**1. Communication models and health IT functions**

The integration of health IT tools within various communication models has been identified, specifically on shared decision-making and patient-centered communication. Tools such as decision aids [26], interactive health communication systems [28], and patient portals [30] have been instrumental in increasing patient participation, bridging communication gaps, and improving access to health information [29]. Additionally, tools such as EHRs and health risk assessment instruments have helped in managing health information effectively [34], ensuring that care is tailored to individual patient needs.

**2. Challenges and barriers**

Studies have revealed significant challenges in the integration of health IT, such as technological integration issues [30,34], resistance from health care providers due to the perceived increase in their workload [26,29], lack of personalized help [31], and a wide range of patient technology *literacy* [28,29]. *These challenges demand focused attention on design and usability improvements to promote user engagement and acceptance of health IT solutions.*

**3. Better practices and recommendations**

The literature suggests ongoing training and education for both patients and health care providers as essential [26,30]. It also recommends designing health IT tools that are user-friendly and adaptable to various patient needs [28,29]. Moreover, integrating these tools into clinical workflows is vital to ensure they support rather than disrupt the patient-care provider relationship [34].

Future research should conduct a detailed examination of the specific challenges and opportunities highlighted in these studies to enhance patient engagement and address potential issues. Further efforts should focus on refining health IT tools to facilitate shared decision-making and patient-centered care, ensuring that the implementation of these technologies effectively meets the diverse needs of both patients and physicians, particularly in supporting their interactions. Such targeted research is fundamental for enhancing our understanding of how health IT can effectively assist in patient-physician interactions.

## Discussion

### Principal Findings

The rapid expansion of this topic has generated a substantial data set, enabling us to identify 3 key themes that are essential for integration into the health communication model. In this section, we further investigate how this extensive review enhances our understanding of the critical topics and factors that influence health communication between patients and physicians in the digital era. We also explain how the trends and interactions among these key elements such as health IT, patient empowerment, trust, and patient-physician relationships are reshaping health communication. Additionally, we further elaborate on the significant role and impact of health IT as derived from our analysis. Finally, we outline potential future research opportunities, especially with technology that could further advance this field.

### Primary Topics in Health Communication Models

The evolution of health communication models reflects a clear transition from physician-centered to patient-centered approaches. Earlier models primarily focused on the unidirectional flow of information from physicians to patients [54]. However, with the increasing emphasis on patient empowerment, engagement, and shared decision-making, more recent models prioritize collaboration between patients and physicians, ensuring that medical decisions are aligned with patients’ values and preferences [55]. Patient-centered care and shared decision-making models have been identified in previous studies that encourage active engagement of patients, emphasizing the importance of clear, transparent, and effective communication between patients and physicians [2]. However, the integration of essential elements in health communication models was not clearly elaborated, especially in the digital age where health IT should be effectively utilized in patient-physician communication.

Our study identified 3 primary topics that should be considered to include in a refined health communication model that fits today’s digital age and more collaborative, patient-centered approaches. Specifically, topic 1 underscores the prominence of the Patient-Physician Relationship and Shared Decision-Making in health communication, closely aligning with the collaborative environment and further emphasizing the importance of the patient-physician relationship. Topic 2 captures the rise of Patient Empowerment and Education Strategies. As patients are increasingly empowered to actively engage with their health information, carefully designed education strategies can help effectively bridge the knowledge gap between patients and physicians and avoid patients being misinformed [56]. Topic 3 highlights the broader Health Care Systems and Health IT Implementations that support these communication models. Existing health IT tools such as patient portals, EHRs, and online information systems facilitate patient engagement and streamline communication across the care continuum. However, the integration of these tools into communication models requires careful consideration to ensure both general applications and personalized utilizations.

Addressing these elements can better align health communication models with the evolving needs of health care systems, ensuring that patient empowerment and engagement remain central. Future research should focus on standardizing these models with the integration of these primary topics to ensure they remain flexible and relevant in a rapidly changing health care landscape.

### Trend Analysis and Factor Associations in Health Communication

In our trend analysis, we not only investigated the trend of identified primary topics in topic modeling, but also explored the complex interactions among carefully selected secondary topics, including patient empowerment, health IT, patient-physician relationship, and trust.

Topic 1 (Patient-Physician Relationship and Shared Decision-Making) combined 2 themes of patient-physician relationship and shared decision-making, emphasizing the critical role of strong interpersonal relationships in shared decision-making and collaborations in health care. Notably, a peak in this topic (Figure 3, green) observed in 2021 indicates an increased focus on fostering patient-physician relationships and facilitating collaborative decision-making. This shift was likely influenced by the global health challenges and the push for more patient-centered care models [55]. The critical need for effective communication between patients and physicians emphasized the importance of involving patients more actively in their care decisions and building strong patient-physician relationships, specifically with the support of technology [57]. For instance, the expansion of remote care and digital health technologies has effectively addressed barriers such as distance, waiting times, and disparities in access to care [58], further facilitating this trend toward improved patient-physician interactions.

For topic 2 (Patient Empowerment and Education Strategies), an early and consistent upward trend has been observed in Figure 3 (orange), indicating a shift toward empowering patients with knowledge and tools to manage their health effectively. This trend aligns with broader health care movements that emphasize increased patient education and empowerment. Research has demonstrated that well-implemented education and empowerment strategies can significantly reduce hospital readmission rates and improve adherence to treatment plans [56]. Moreover, higher levels of patient activation are associated with lower health care costs and better outcomes [59]. However, the journey to effectively empower patients continues to evolve. Supporting this evolution, effective health communication between patients and physicians plays an essential role in the empowerment process [60]. Enhanced communication fundamentally supports patient empowerment by providing clearer, more accessible information, which is essential for informed decision-making. Therefore, it is crucial to develop and carefully integrate patient empowerment strategies within health communication frameworks. This integration ensures that communications not only inform but also actively engage patients, thereby enhancing their role in decision-making processes and overall care management to reach better health care outcomes.

For topic 3 (Health Care Systems and Health IT Implementations), the significant peaks observed in 2016 and 2023 correspond to critical periods of health care system reforms (Figure 3, blue), particularly those involving the integration of new technologies. The enhanced use of health IT such as EHRs and web-based communication tools facilitated improved patient involvement and personalized care [34,35,37]. The COVID-19 pandemic further accelerated the implementation of advanced digital tools such as health communicator, which were designed to meet specific patient needs, including tailored information and psychosocial support for individuals [51,52]. This reflects a field that is continually adapting, not only to technological advancements but also to the evolving needs of patients. These developments illustrate the dynamic nature of health communication research and highlight critical moments when topics such as patient empowerment, shared decision-making, and technology integration rise to prominence.

Additional analyses involved refined secondary terms that helped us to investigate the detailed associations between specific terms that represented the core idea of the primary topics. This targeted selection process was crucial for accurately mapping the evolution of specific themes within health communication over the study period, enabling us to uncover nuanced trends and associations that might be unnoticed in a broader topic modeling analysis.

The top 2 strongest intersections we explored emphasize the critical role of the patient-physician relationship in health communication, particularly as patients take more control in the context of patient-centered care [61]. The integration of patient empowerment within the intersections highlights the necessity of bridging the knowledge gap for patients, enabling them to engage more actively in their health care decisions. Additionally, the third most prominent intersection, involving health IT, further underscores the growing role of technology in empowering patients and facilitating effective communication. Although the topic of trust appeared less frequently in association with other secondary terms, its close connections with each term were consistently identified. These individual connections emphasize the multifaceted role that trust plays in health communication, demanding further investigation. Specifically, trust acts as a foundational element in promoting better treatment adherence and fostering stronger patient-physician relationships [62]. Moreover, trust in health IT is equally vital, influencing patients’ willingness to adopt and engage with digital health solutions, thereby enhancing the overall effectiveness of health communication [45,63].

### The Role of Health IT in Health Communication Models

In our in-depth synthesis and analysis (shown in Table 2), many communication-related technologies are not under any specific communication model, but only guided by the idea of shared decision-making or facilitating patients’ participation. Previous studies suggested that while shared decision-making is widely endorsed, many implementations lack a structured model that consistently leads to effective communication outcomes [64,65]. Similarly, patient-centered communication, despite its widespread advocacy as a concept that should lead to more satisfactory health care processes and outcomes, often remains theoretical rather than practical in its application [66]. Despite these challenges in the implementation of shared decision-making and patient-centered communication, the setting began to change significantly with technological advancements. By 2016, significant advancements in health care were driven by the adoption of digital solutions, addressing key shortcomings of traditional communication methods, such as inefficiency in data management, limited patient access, and fragmented communication [30,67]. Health IT, particularly through web-based interventions, began to enhance the structural foundation and accessibility of health care communications. Specifically, the integration of EHRs with patient portals has matured significantly, enhancing communication channels between patients and health care providers [34,68]. Additionally, the implementation of web-based communication tools, decision aids, and online learning platforms has significantly advanced patient education, engagement, and effective communication with physicians [34-37]. These technologies provide essential continuity in patient care and enhance direct interactions between health care providers and patients, fundamentally altering the efficiency and effectiveness of health care delivery. By 2021, the COVID-19 pandemic had further catalyzed design improvements and validations for the application of health IT. The development of digital health, such as conversational aids, emerged as a notable advancement during this period. These tools, essential in a time of increased remote interactions due to social distancing measures, are designed to enhance patient engagement and support shared decision-making processes [46,47]. They aim to provide personalized, accessible information that patients can easily navigate and understand, filling a critical gap as in-person health care interactions become less frequent [45,48,51]. This pandemic emphasized the importance of reliable, accessible health communication technologies, driving innovations that could meet the new health care demands efficiently and effectively.

Building on the significant progress made in health IT, the evolution of health communication has been deeply intertwined with technological advancements across multiple dimensions. These advancements include facilitating interactions between patients and physicians, managing health care data, enhancing education, and improving overall health care delivery [68]. However, despite these advances, challenges and barriers persist in integrating human interactions with health IT systems. Notably, aspects such as the patient-physician relationship and trust building remain underexplored, despite being crucial for optimizing the effectiveness of health communication [39-42]. Addressing these gaps is essential to ensure that health IT supports not only the operational but also the interpersonal aspects of health care delivery, paving the way for more comprehensive and effective communication strategies.

### Future Outlook: Artificial Intelligence in Health Communication

AI is transforming patient-physician communication by facilitating more personalized, efficient, and informed interactions through advanced data analysis and real-time information processing. AI is also increasingly being utilized to support or inform health care diagnoses and decisions [69,70]. Emerging AI tools, such as ChatGPT and other generative AI systems, analyze vast amounts of written material to generate coherent, contextually relevant text that closely resembles human writing styles. The use of AI before patient-physician interactions can significantly reshape the traditional dynamics of knowledge and power. Traditionally, physicians, with years of training and experience, have held professional knowledge, guiding the flow and outcomes of health care discussions. However, AI technologies—particularly those offering instant access to vast medical information—empower patients with a form of “pocket expertise.” This access enables patients to approach consultations with a preliminary understanding of potential diagnoses, treatments, and even novel therapies. Such information can help level the playing field, fostering a more collaborative relationship. However, it may also introduce tension or conflict between patients and health care professionals. While AI can rapidly process and synthesize information, it can also produce incorrect or misleading conclusions [71]. The technology’s interpretations are only as reliable as the data and algorithms driving them, which may lack the nuanced or contextual understanding of a health care professional.

This revolution in patient-physician communication through AI has the potential to transform interaction dynamics, offering both opportunities and challenges that warrant further exploration. Future studies should investigate AI’s potential to enhance personalized medical knowledge and its implications for health care decision-making, while carefully addressing the limitations of these technologies.

### Limitations

The analysis of associations and trends in our study offers valuable insights into the dynamics of health communication influenced by health IT, patient empowerment, and patient-physician relationships, as well as the interactions among these key factors. However, it is important to emphasize that these associations should not be interpreted as direct causal relationships. Our analysis primarily identifies correlations that suggest potential interactions between these variables, providing a foundation for future hypotheses and more in-depth research tailored to specific situations and conditions. The identification of these patterns is essential for developing a more nuanced understanding of how various factors contribute to the effectiveness of health communication. This understanding can then inform subsequent experimental studies aimed at verifying causality and uncovering the underlying mechanisms at play.

Our review focused broadly on the general interactions between patients and physicians across diverse health care settings. As a result, specific demographic variables—such as different diseases, age groups, and racial backgrounds—were not extensively differentiated in our analysis. This broad approach aligns with our goal of identifying overarching trends and commonalities in health communication. However, it is acknowledged that health communication dynamics can vary significantly across different patient groups, shaped by unique needs and contexts related to specific health conditions, age-related preferences, and cultural or racial backgrounds. Future research could benefit from stratifying these variables to gain more detailed insights and develop communication strategies that are better tailored to the specific characteristics and needs of diverse patient populations.

Finally, this paper presents several important findings, such as the interactions between key terms identified in our trend analysis. However, the depth of synthesis regarding these intersections remains limited. Our synthesis primarily focuses on elucidating the role of health IT in health communication, highlighting its significant applications across diverse health care contexts. While the study offers a broad overview, outlining relevant terms and setting the stage for future research, it leaves room for more focused investigations. Subsequent studies are encouraged to investigate each finding in greater detail, further exploring the complex dynamics at play and refining our understanding of these critical intersections in health communication.

### Conclusions

This review extensively explored the key factors influencing health communication between patients and physicians in the digital age. Our analysis identified 3 main topics central to the field: the Patient-Physician Relationship and Shared Decision-Making, Patient Empowerment and Education Strategies, and Health Care Systems and Health IT Implementations. These topics serve as foundational elements for refining health communication models that foster effective interactions between patients and physicians. Additionally, this review highlighted significant interactions between health IT and key aspects of health communication, particularly patient-physician relationships, patient empowerment, and trust. The COVID-19 pandemic accelerated the adoption of digital health technologies such as telehealth, patient portals, and decision aids, all of which have demonstrated great potential in enhancing these interactions. Finally, the synthesis of health IT’s role in health communication reveals its transformative impact on patient empowerment and engagement, while also highlighting significant challenges related to integration, usability, and user acceptance. These findings emphasize the need for continuous refinement of health IT tools to better support patient-centered care and informed decision-making within health communication. Future research should focus on developing models that integrate health IT and foster trust-based relationships between patients and physicians. Investigating how emerging technologies, such as AI, can enhance these models will also be crucial. Such efforts are essential for realizing the full potential of health IT in improving health care outcomes through effective communication between patients and physicians.

## Appendix

Multimedia Appendix 1 Details of searching results from 1999 to 2023.

Multimedia Appendix 2 Intertopic distance map and top 30 relevant terms for topics 2 and 3.

## Abbreviations

AI: artificial intelligence
EHR: electronic health record
health IT: health information technology
MeSH: Medical Subject Headings
mHealth: mobile health
PICo: Population, Interest, and Context
