# Supplementary material for: Digital Age Transformation in Patient-Physician Communication: 25-Year Narrative Review (1999-2023)
Source: J Med Internet Res. 2025 Jan 16;27:e60512. doi: 10.2196/60512 (PMC11783030; doi:10.2196/60512)
Supplement: Multimedia Appendix 1 [file jmir_v27i1e60512_app1.docx]

### **Appendix 1:** Details of Searching Results from 1999 to 2023

| **Key Terms** | **MeSH Terms** | **Database** | **Searching Results** |
| --- | --- | --- | --- |
| *((communication) OR (health communication model)) AND ((decision making*) OR (decision making, shared)) AND ((patient participation*) OR (patient participation/methods) OR (patient empowerment) OR (patient engage*) OR (patient involve*) OR (Patient-Centered Care)) AND ((physician-patient relations*) OR (Trust) OR (physician-patient trusted relationship)) AND ((decision support techniques) OR (technology) OR (health informatics)* *OR (Patient Preference) OR (information systems*) OR (health information systems))* Filters: from 1999 - 2023 | "communication"[MeSH Terms]  "health communication"[MeSH Terms]  "decision making, shared"[MeSH Terms]  "patients"[MeSH Terms]  "patient participation"[MeSH Terms]  "patient-centered care"[MeSH Terms]  "physician-patient relations"[MeSH Terms]  "trust"[MeSH Terms]  "decision support techniques"[MeSH Terms]  "technology"[MeSH Terms]  "medical informatics"[MeSH Terms]  "patient preference"[MeSH Terms]  "health information systems"[MeSH Terms] | PubMed | 779 |
| (((health communication model) OR (health communication)) AND ((shared decision making) OR (patient participation) OR (patient empowerment) OR (patient engage) OR (patient involve) OR (Patient-Centered Care)) AND ((decision support techniques) OR (technology) OR (health informatics) OR (information systems) OR (health information systems)))  Filters: from 1999 - 2023 | "health communication"[MeSH Terms]  "decision making, shared"[MeSH Terms]  "patients"[MeSH Terms]  "patient participation"[MeSH Terms]  "social participation"[MeSH Terms]  "patient-centered care"[MeSH Terms]  "decision support techniques"[MeSH Terms]  "technology"[MeSH Terms]  "information systems"[MeSH Terms]  "health information systems"[MeSH Terms] | IEEE Xplore | 1022 |
| ALL=(("Health Communication" OR "health communication models" OR "communication") AND ("Patient Participation" OR "patient empowerment" OR "patient engagement" OR "patient involvement" OR "patient-centered" ) AND ("Decision Making, Shared" OR "shared decision making" OR "Physician-Patient Relations" OR "patient-physician relationship" OR "trust relationship") AND ("Telemedicine" OR "digital health" OR "eHealth" OR "mHealth" OR "Telehealth" OR "Electronic Health Records" OR "EHR" OR "technology"))  **Note**: includes results from 2008 to 2023 (no data available before 2008) | "communication"[MeSH Terms]  "health communication"[MeSH Terms]  "decision making, shared"[MeSH Terms]  "patients"[MeSH Terms]  "patient participation"[MeSH Terms]  "patient-centered care"[MeSH Terms]  "physician-patient relations"[MeSH Terms]  "trust"[MeSH Terms]  "decision support techniques"[MeSH Terms]  "technology"[MeSH Terms]  "medical informatics"[MeSH Terms]  "health information systems"[MeSH Terms] | Web of Science | 113 |
| ( ( health AND communication AND model ) ) AND ( decision AND making, AND shared ) AND ( ( patient AND participation ) OR ( patient AND empowerment ) OR ( patient AND engage ) OR ( patient-centered AND care ) ) AND ( ( physician-patient AND relations ) OR ( trust ) ) AND ( health AND information AND systems ) AND PUBYEAR > 1998 AND PUBYEAR < 2024 AND ( LIMIT-TO ( SRCTYPE , "j" ) ) AND ( LIMIT-TO ( OA , "all" ) ) AND ( LIMIT-TO ( PUBSTAGE , "final" ) ) AND ( LIMIT-TO ( DOCTYPE , "ar" ) ) AND ( LIMIT-TO ( LANGUAGE , "English" ) ) | "communication"[MeSH Terms]  "health communication"[MeSH Terms]  "decision making, shared"[MeSH Terms]  "patients"[MeSH Terms]  "patient participation"[MeSH Terms]  "patient-centered care"[MeSH Terms]  "physician-patient relations"[MeSH Terms]  "decision support techniques"[MeSH Terms]  "technology"[MeSH Terms]  "medical informatics"[MeSH Terms]  "health information systems"[MeSH Terms] | Scopus | 1,246 |
| ( ( health AND communication ) OR ( health AND communication AND model ) ) AND ( decision AND making, AND shared ) AND ( ( patient AND participation* ) OR ( patient AND empowerment ) OR ( patient AND engage* ) OR ( patient-centered AND care ) ) AND ( ( physician-patient AND relations* ) ) AND ( ( technology ) OR ( information AND systems* ) OR ( health AND information AND systems ) )  **Note**: with open access and smartext searching function in APA PsycInfo | "communication"[MeSH Terms]  "health communication"[MeSH Terms]  "decision making, shared"[MeSH Terms]  "patients"[MeSH Terms]  "patient participation"[MeSH Terms]  "patient-centered care"[MeSH Terms]  "physician-patient relations"[MeSH Terms]  "decision support techniques"[MeSH Terms]  "technology"[MeSH Terms]  "medical informatics"[MeSH Terms]  "health information systems"[MeSH Terms] | PsycINFO | 231 |
|  |  |  |  |
| Total: |  |  | 3,391 |
