# Supplementary figures and images for: Digital Age Transformation in Patient-Physician Communication: 25-Year Narrative Review (1999-2023)
Source: J Med Internet Res. 2025 Jan 16;27:e60512. doi: 10.2196/60512 (PMC11783030; doi:10.2196/60512)

### **Appendix 2.** Intertopic distance map and top 30 relevant terms for Topic 2 and Topic 3


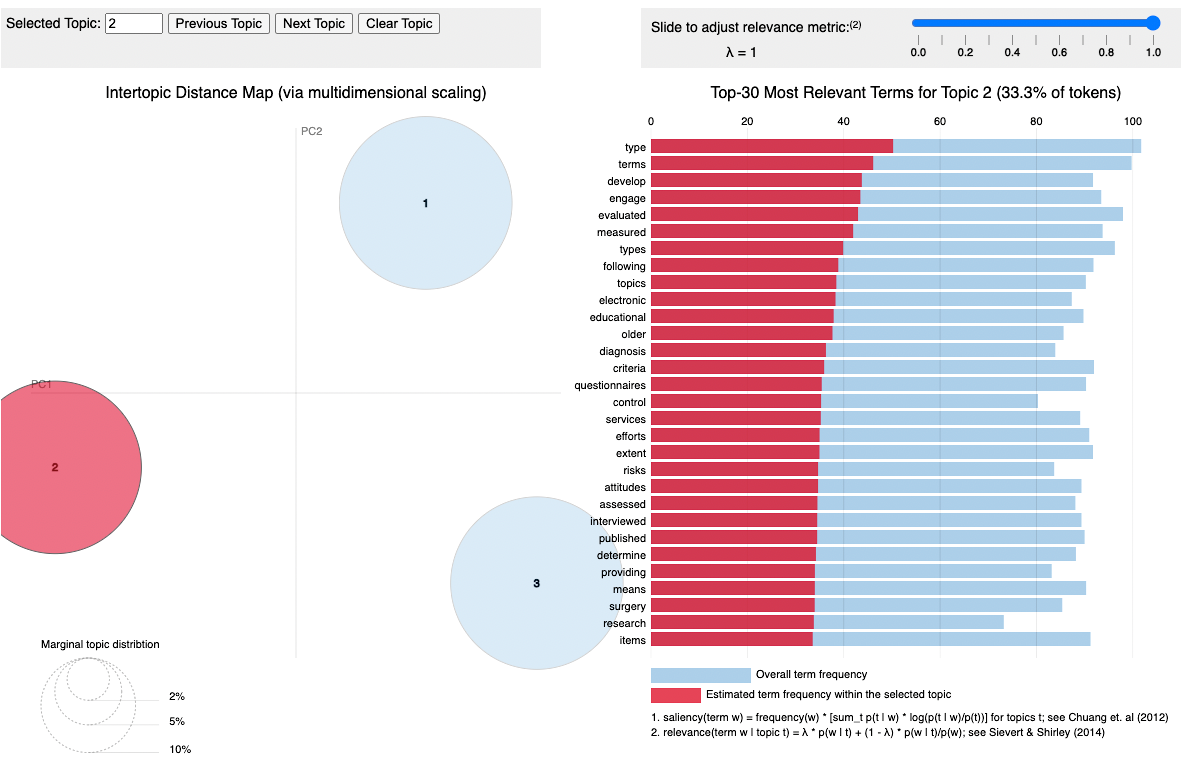


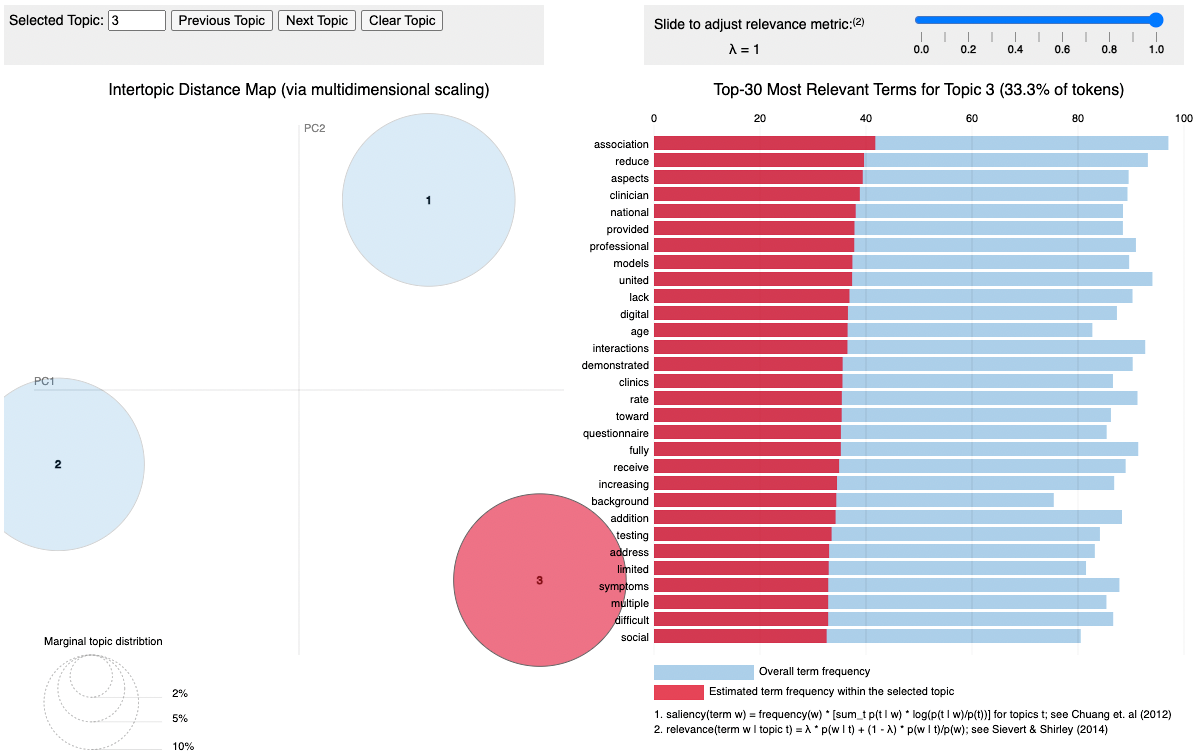

Supplement: Multimedia Appendix 2 [file jmir_v27i1e60512_app2.docx]
